# Supplementary material for: Foliar Lesions Induced by Pestalotiopsis arengae in Oil Palm (O × G) in the Colombian Southwest Palm Zone
Source: J Fungi (Basel). 2023 Dec 29;10(1):24. doi: 10.3390/jof10010024 (PMC10817598; doi:10.3390/jof10010024)
Supplement: Supplementary file 1 [file jof-10-00024-s001.zip › jof-2641372-supplementary.pdf]

Journal of Fungi

Supplementary Materials:

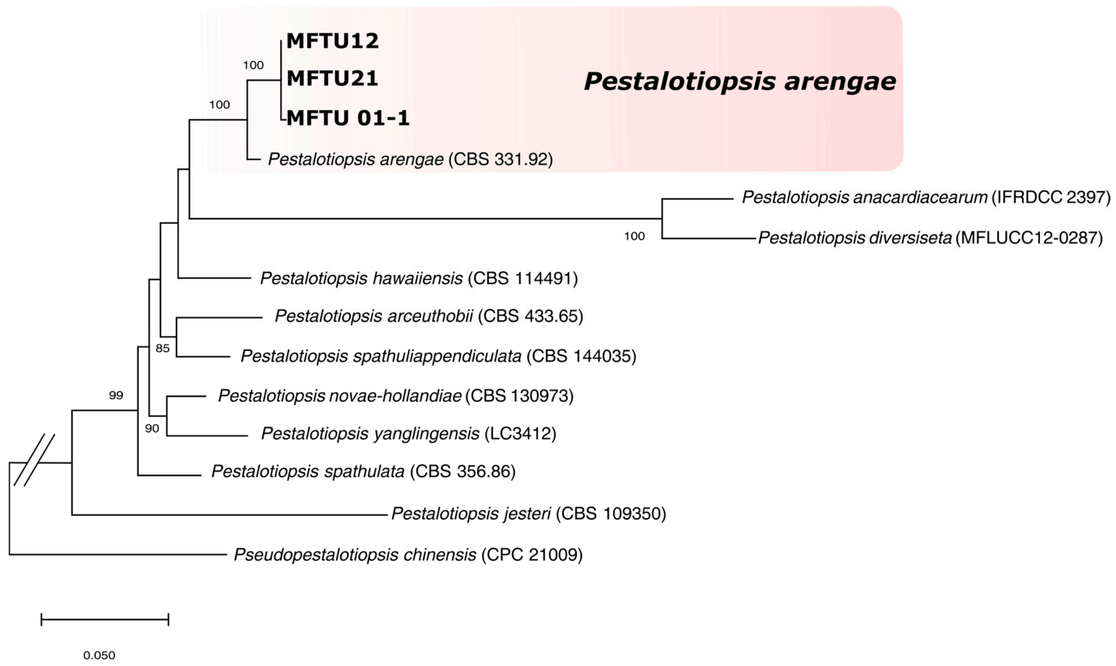

Supplementary Figure S1. Phylogenetic tree obtained via maximum likelihood estimation of the consensus of the *ITS* region of some species of the genus *Pestalotiopsis*. The specie *Pseudopestalotiopsis chinensis* (CPC 21009) was included as an outgroup.

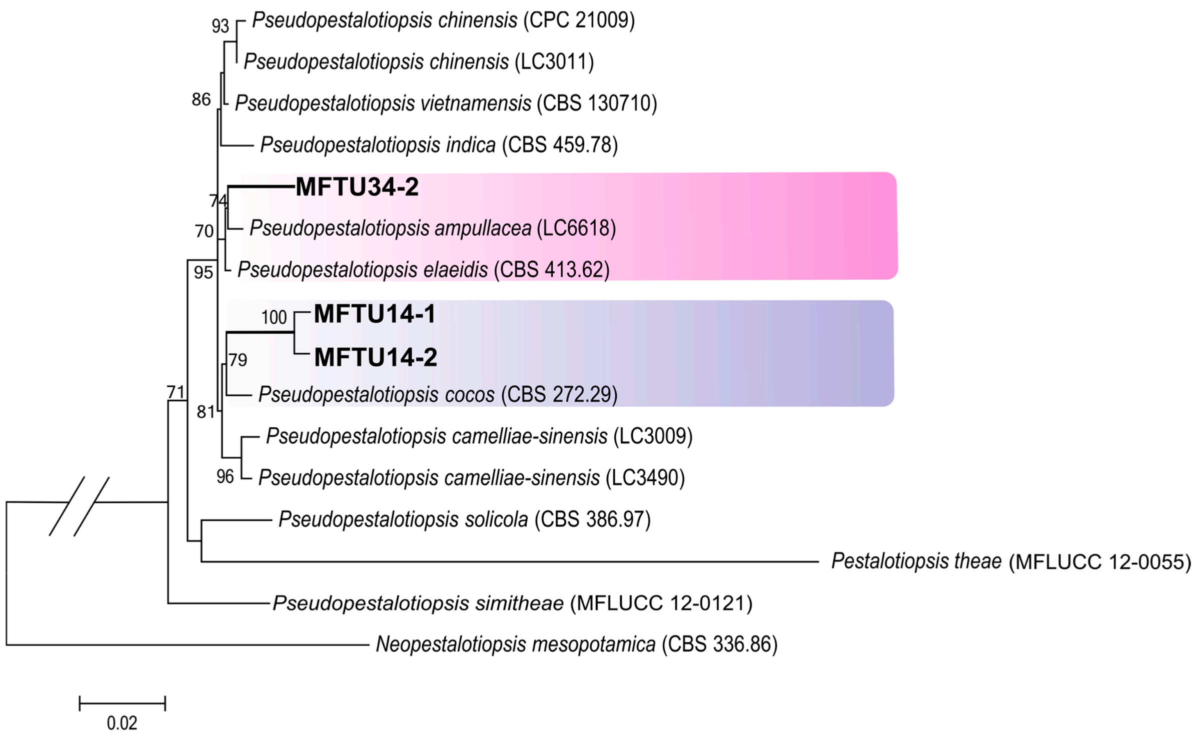

Supplementary Figure S2. Phylogenetic tree obtained via maximum likelihood estimation of the consensus of the *ITS* region of some species of the genus *Pseudopestalotiopsis*. The specie *Neopestalotiopsis mesopotamica* (CBS 336.86) was included as an outgroup.

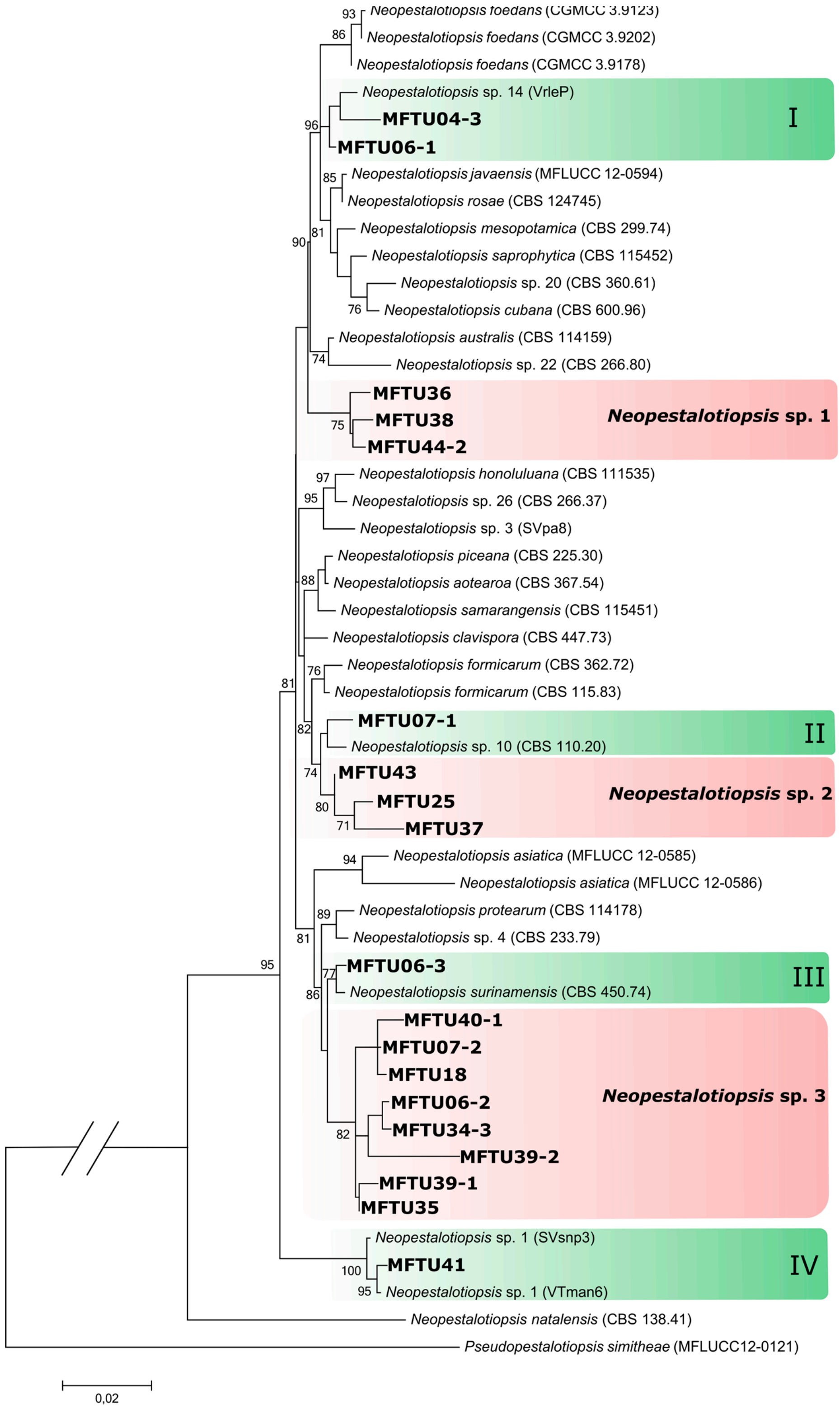

Supplementary Figure S3. Phylogenetic tree obtained via maximum likelihood estimation of the consensus of the *ITS* region of some species of the genus *Neopestalotiopsis*. The specie *Pseudopestalotiopsis simithae* (MFLUCC12-0121) was included as an outgroup.

Table S1: Isolate codes, taxonomic identification, and accession numbers reported in the GenBank used in this study.

Strain/Isolate	Taxon	GenBank Accession Number	Reference
**CBS 433.65**	*Pestalotiopsis arceuthobii*	MH554046.1	F. Liu, 2019. *Sporocadaceae*, a family of coelomycetous fungi with appendage-bearing conidia.
**CBS:433.65**	*P. maculiformans*	MH858655.1	Vu D., 2019. Large-scale generation and analysis of filamentous fungal DNA barcodes boosts coverage for kingdom fungi and reveals threshold for fungal species and higher taxon delimitation.
**CBS 331.92**	*P. arengae*	KM199340.1	Maharachchikumbura et al., 2014. *Pestalotiopsis* revisited.
**SAUCC230866**	*P. portugallica*	OQ692014	Liu S., 2023. Direct submission.
**SAUCC230867**	*P. portugallica*	OQ692015.1	Liu S., 2023. Direct submission.
**CBS:236.38**	*P. biciliata*	MK862235.1	Vu D., 2019. Large-scale generation and analysis of filamentous fungal DNA barcodes boosts coverage for kingdom fungi and reveals threshold for fungal species and higher taxon delimitation.
**CBS 790.68**	*P. biciliata*	KM199305	Maharachchikumbura et al., 2014. *Pestalotiopsis* revisited.
**CBS:171.26**	*P. oryzae*	MH854881.1	Vu D., 2019. Large-scale generation and analysis of filamentous fungal DNA barcodes boosts coverage for kingdom fungi and reveals threshold for fungal species and higher taxon delimitation.
**MWN35**	*P. oryzae*	OM899910	Waweru B.N. and Mutiga S., 2022. Direct submission.
**CBS:176.25**	*P. scoparia*	MH854838.1	Vu D., 2019. Large-scale generation and analysis of filamentous fungal DNA barcodes boosts coverage for kingdom fungi and reveals threshold for fungal species and higher taxon delimitation.
**BGTU**	*P. scoparia*	MT186821.1	Panteleev S. and Baranov O., 2022. Direct submission.
**CBS 186.71**	*P. chamaeropis*	KM199326.1	Maharachchikumbura et al., 2014. *Pestalotiopsis* revisited.
**CBS 113607**	*P. chamaeropis*	KM199325	Maharachchikumbura et al., 2014. *Pestalotiopsis* revisited.
**CBS 237.38**	*P. chamaeropis*	KM199324.1	Maharachchikumbura et al., 2014. *Pestalotiopsis* revisited.
**CBS 331.96**	*P. papuana*	NR_147553.1	Maharachchikumbura et al., 2014. *Pestalotiopsis* revisited.
**CBS 887.96**	*P. papuana*	KM199318	Maharachchikumbura et al., 2014. *Pestalotiopsis* revisited.
**CBS 115585**	*P. diploclisia*	KM199315.1	Maharachchikumbura et al., 2014. *Pestalotiopsis* revisited.
**CBS 115449**	*P. diploclisia*	KM199314	Maharachchikumbura et al., 2014. *Pestalotiopsis* revisited.
**SAUCC230471**	*P. diploclisia*	OQ691988	Liu S., 2023. Direct submission.
**CBS 336.97**	*P. humus*	KM199317.1	Maharachchikumbura et al., 2014. *Pestalotiopsis* revisited.
**CBS 115450**	*P. humus*	KM199319	Maharachchikumbura et al., 2014. *Pestalotiopsis* revisited.
**CBS 111503**	*P. australis*	KM199331.1	Maharachchikumbura et al., 2014. *Pestalotiopsis* revisited.
**CBS 114474**	*P. australis*	KM199334	Maharachchikumbura et al., 2014. *Pestalotiopsis* revisited.
**CBS 119350**	*P. australis*	KM199333.1	Maharachchikumbura et al., 2014. *Pestalotiopsis* revisited.
**CBS:357.71**	*P. neglecta*	MH860161.1	Vu D., 2019. Large-scale generation and analysis of filamentous fungal DNA barcodes boosts coverage for kingdom fungi and reveals threshold for fungal species and higher taxon delimitation.
**LPS-23**	*P. neglecta*	KX355189.1	Li L., Pan H., and Zhon C., 2016. Direct submission.
**LWJ86**	*P. neglecta*	MN218809.1	Padamsee M. and Burgess B.M., 2019. Direct submission.
**LC6618**	*Pseudopestalotiopsis ampullacea*	KX895025.1	F. Liu, 2017. Pestalotiopsis and allied genera from Camellia, with description of 11 new species from China.
**TXYLBJ6**	*Ps. camelliae-sinensis*	MN198157.1	Liu, 2019. Identification and pathogenicity analysis of pathogenic fungi of tea plant.
**CLBB1**	*Ps. camelliae-sinensis*	MK909901.1	Liu, 2019. Identification and pathogenicity analysis of pathogenic fungi of tea plant.
**TXYLBJ4**	*Ps. camelliae-sinensis*	MN198155.1	Liu, 2019. Identification and pathogenicity analysis of pathogenic fungi of tea plant.
**NTUCC 18-066**	*Ps. chinensis*	MT322083.1	Tsai I. and Ariyawansa H.A., 2020. Direct Submission.
**NTUCC 18-061**	*Ps. chinensis*	MT322078.1	Tsai I. and Ariyawansa H.A., 2020. Direct Submission.
**NTUCC 18-056**	*Ps. chinensis*	MT322073.1	Tsai I. and Ariyawansa H.A., 2020. Direct Submission.
**CBS 272.29**	*Ps. cocos*	NR_145246	Maharachchikumbura et al., 2014. *Pestalotiopsis* revisited.
**CZ-4**	*Neopestalotiopsis chrysea*	MT459336.1	Wu F., 2020. Direct Submission.
**MFLUCC 12-0585**	*N. asiatica*	KX816923.1	Maharachchikumbura, 2017. Characterization of *Neopestalotiopsis*, *Pestalotiopsis*, and *Truncatella* species associated with grapevine trunk diseases in France.
**CBS 114159**	*N. australis*	KM199348.1	Maharachchikumbura et al., 2014. *Pestalotiopsis* revisited.
**MFLUCC 17-1555**	*N. brachiata*	MK764274.1	Norphanphoun C. et al., 2019. Morphological and phylogenetic characterization of novel pestalotiopsis species associated with mangroves in Thailand.

**CBS 447.73**	*N. clavispora*	MH860736.1	Vu D., 2019. Large-scale generation and analysis of filamentous fungal DNA barcodes boosts coverage for kingdom fungi and reveals threshold for fungal species and higher taxon delimitation.
**YZM-1**	*N. clavispora*	OM721785.1	Lin Q., 2022. Direct Submission.
**CBS 600.96**	*N. cubana*	KM199347.1	Maharachchikumbura et al., 2014. *Pestalotiopsis* revisited.
**KACC48638**	*N. foedans*	MK396465.1	Park H. and Eom A., 2019. Direct Submission.
**COUFAL0244**	*N. foedans*	MT605379.1	Barbosa T.J.A et al., 2020. First report of *Neopestalotiopsis foedans* causing pestalotia spot in leaf on coconut palm in Brazil, Barbosa.
**CBS 115.83**	*N. formicarum*	KM199344.1	Maharachchikumbura et al., 2014. *Pestalotiopsis* revisited.
**JSNJ6B**	*N. formicarum*	MW578369.1	Liu C., 2021. First Report of Leaf Spot Disease Caused by *Neopestalotiopsis formicarum* on Acer negundo.
**ZHKUCC:22-0114**	*N. fragariae*	ON651145.1	Prematunga C.J. et al., 2022. Addition to pestalotioid fungi in China: *Neopestalotiopsis fragariae* sp. nov. causing leaf spots on Fragaria x ananassa.
**CBS 257.31**	*N. javaensis*	MH855207.1	Vu D., 2019. Large-scale generation and analysis of filamentous fungal DNA barcodes boosts coverage for kingdom fungi and reveals threshold for fungal species and higher taxon delimitation.
**CBS 111535**	*N. honoluluana*	KM199363.1	Maharachchikumbura et al., 2014. *Pestalotiopsis* revisited.
**MFLUCC 22-0149**	*N. hydeana*	OP802386.1	Seifollahi E., 2023.Taxonomic Advances from Fungal Flora Associated with Ferns and Fern-like Hosts in Northern Thailand.
**CBS 299.74**	*N. mesopotamica*	KM199361.1	Maharachchikumbura et al., 2014. *Pestalotiopsis* revisited.
**CBS 336.86**	*N. mesopotamica*	NR_145244.1	Maharachchikumbura et al., 2014. *Pestalotiopsis* revisited.
**BRIP 66617**	*N. nebuloides*	OM417249.1	Steinrucken T.V. and Tan Y.P., 2020. Diversity of fungal species associated with Clade A Sporobolus species in Queensland, Australia.
**BRIP 71166**	*N. nebuloides*	OM417308.1	Steinrucken T.V. and Tan Y.P., 2020. Diversity of fungal species associated with Clade A Sporobolus species in Queensland, Australia.
**MFLUCC 17-1738**	*N. petila*	MK764276.1	Norphanphoun C. et al., 2019. Morphological and phylogenetic characterization of novel pestalotioid species associated with mangroves in Thailand.
**CBS 225.30**	*N. piceana*	MH855130.1	Vu D., 2019. Large-scale generation and analysis of filamentous fungal DNA barcodes boosts coverage for kingdom fungi and reveals threshold for fungal species and higher taxon delimitation.
**CBS 254.32**	*N. piceana*	KM199372.1	Maharachchikumbura et al., 2014. *Pestalotiopsis* revisited.
**CBS 394.48**	*N. piceana*	KM199368.1	Maharachchikumbura et al., 2014. *Pestalotiopsis* revisited.
**MFLUCC 22-0157**	*N. phangngaensis*	OP802390.1	Seifollahi E., 2023. Taxonomic Advances from Fungal Flora Associated with Ferns and Fern-like Hosts in Northern Thailand.
**CBS 114178**	*N. protearum*	LT853103.1	Bonthond G. et al., 2017. Taxonomy of the phytopathogenic genus *Seiridium* (Sporocadaceae).
**OTTR524**	*N. protearum*	KT936426.1	Monclova-Santana C. et al., 2015. Pathogenic threats of ten endangered plant species of the karst region of Puerto Rico.
**CBS 115451**	*N. samarangensis*	KM199365.1	Maharachchikumbura et al., 2014. *Pestalotiopsis* revisited.
**CBS 115452**	*N. saprophytica*	KM199345.1	Maharachchikumbura et al., 2014. *Pestalotiopsis* revisited.
**CBS 450.74**	*N. surinamensis*	NR_145240.1	Maharachchikumbura et al., 2014. *Pestalotiopsis* revisited.
**MFLUCC 17-1550**	*N. rhizophorae*	MK764278.1	Norphanphoun C. et al., 2019. Morphological and phylogenetic characterization of novel pestalotioid species associated with mangroves in Thailand.
**MFLUCC 17-1551**	*N. rhizophorae*	MK764277.1	Norphanphoun C., 2019. Morphological and phylogenetic characterization of novel pestalotioid species associated with mangroves in Thailand.
**MFLUCC 17-1728**	*N. rhizophorae*	MN871770.1	Hyde K.D., Norphanphoun C., and Maharachchikumbur S.S., 2019. Refined families of Sodariomycetes.
**MFLUCC 17-1745**	*N. sonneratae*	MK764280.1	Norphanphoun C. et al., 2019. Morphological and phylogenetic characterization of novel pestalotioid species associated with mangroves in Thailand.
**MFLUCC 17-1744**	*N. sonneratae*	MK764280.1	Norphanphoun C. et al., 2019. Morphological and phylogenetic characterization of novel pestalotioid species associated with mangroves in Thailand.
**KoRLI047182**	*N. rosae*	MN341550.1	Oh S.Y. et al., 2019. Diversity and distribution patterns of endolichenic fungi in Jeju Island, South Korea.
**JZB340069**	*N. rosae*	MN495977.1	Li Xh and Yan Jy, 2019. Strawberry Root Rot Caused by *Neopestalotiopsis rosae* in China.
**JZB340064**	*N. rosae*	MN495972.1	Li Xh and Yan Jy, 2019. Strawberry Root Rot Caused by *Neopestalotiopsis rosae* in China.
**CBS 124745**	*N. rosae*	KM199360.1	Maharachchikumbura et al., 2014. *Pestalotiopsis* revisited.
**1-22**	*N. vaccinii*	OQ316613.1	Blagojevic J. et al., 2023. Characterization of Fungal Species Associated with Blueberry Dieback in Serbia.
**2-21**	*N. vaccinii*	OQ316612.1	Blagojevic J. et al., 2023. Characterization of Fungal Species Associated with Blueberry Dieback in Serbia.
**KC005788**	*Seiridium phylicae*	NR_158886	Crous P.W. et al., 2012. Fungal Planet description sheets: 107-127.

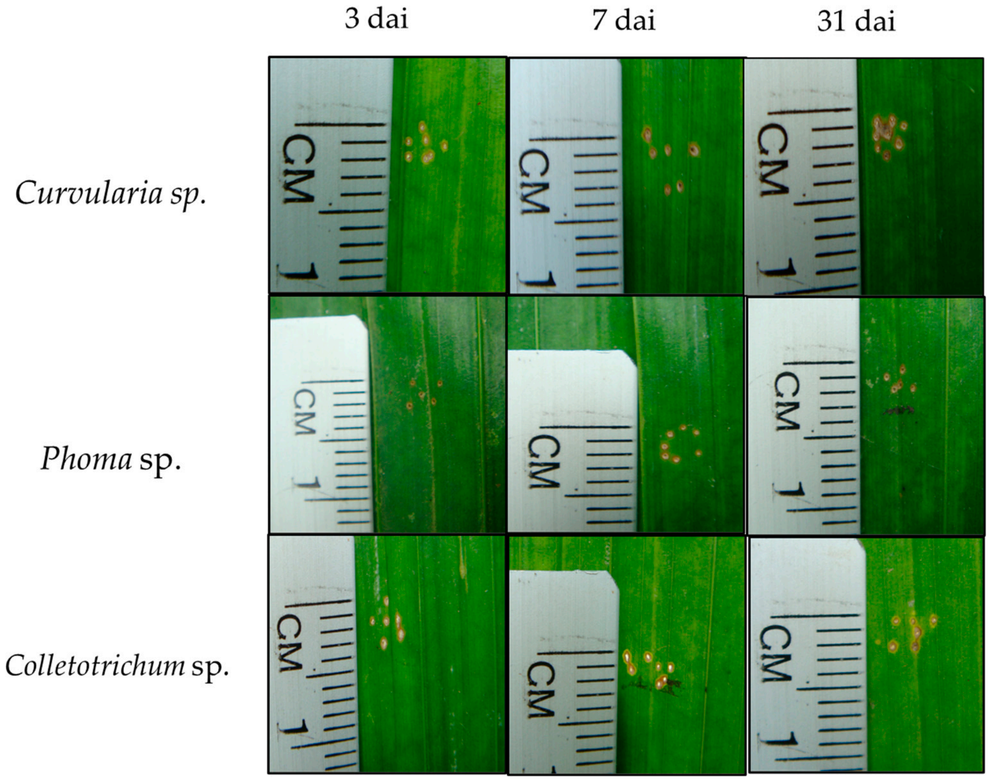

Supplementary Figure S4. Pathogenicity test of *Curvularia* sp., *Phoma* sp., and *Colletotrichum* sp. leaf spot after inoculation, from left to right, at 3, 17, and 31 days after inoculation.

Methodology for inoculation of isolates on other cultivars

To confirm the pathogenicity of the 25 isolates related to *Pestalotiopsis* in other cultivars, palms of the cultivars aged 5-7 months were inoculated: Brazil x 7 pollen africans, Coarí x La Mé and Manaos x Compacta. The inoculation method was the same as that used for the Sinú (cerete) x Deli Yangambi cultivar. A sterile entomological pin (0.40 mm x 37 mm) was used to create the wound in the leaf tissue and 25 mL of a suspension of conidia from each isolate was applied at a concentration of $3x10^6$ conidia/ml (62). Conidia were taken from 10-day-old Petri dishes. The same procedure was used for the control treatment and autoclaved sterile water was used. Measurements were analyzed using ImageJ® software. The photographic record was taken at 21 days. A total of five repetitions were carried out for each isolate, and a palm with four lanceolate leaves with four wounds and inoculation constituted one repetition. Lesion size (mm^2) was measured at 3, 7, and 21 days post-inoculation (dai) and analyzed using repeated measures analysis (RM-ANOVA). Means were analyzed using Tukey's test ($P < 0.05$) using the SAS 9.0 statistical program.

Pathogenicity test of 25 isolates on the Brazil x 7 pollen africans cultivar.

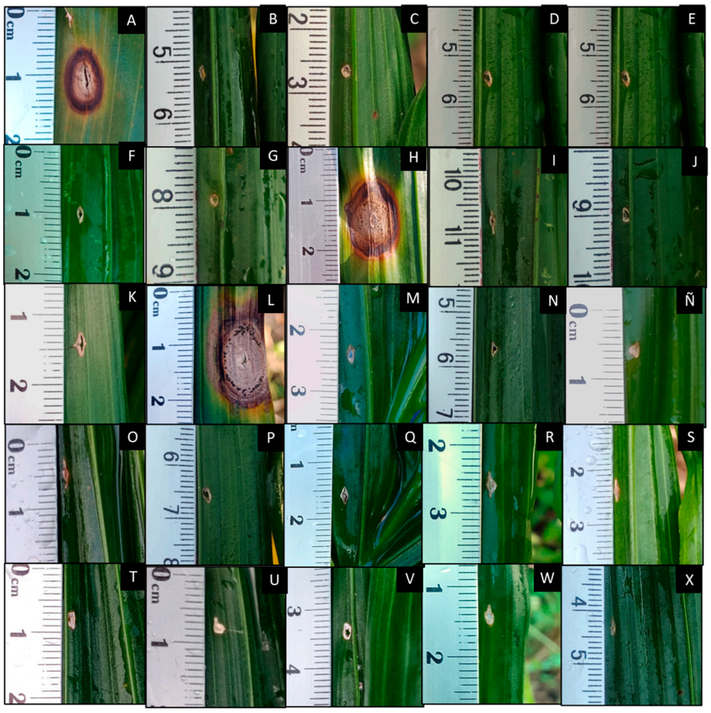

A	MFTU01-1
B	MFTU04-3
C	MFTU06-1
D	MFTU06-2
E	MFTU06-3
F	MFTU07-1
G	MFTU07-2
H	MFTU12
I	MFTU14-1
J	MFTU14-2
K	MFTU18
L	MFTU21
M	MFTU25
N	MFTU34-2
Ñ	MFTU34-3
O	MFTU35
P	MFTU36
Q	MFTU37
R	MFTU38
S	MFTU39-1
T	MFTU39-2
U	MFTU40-1
V	MFTU41
W	MFTU43
X	MFTU44-2

Supplementary Figure S5. Pathogenicity test of the 25 isolates related to *Pestalotiopsis* in cultivar Brazil x 7 pollen africans. The photos were taken 21 days after inoculation, representing isolates A-X as listed in Table 1.

Pathogenicity test of 25 isolates on the Coarí x La Mé cultivar.

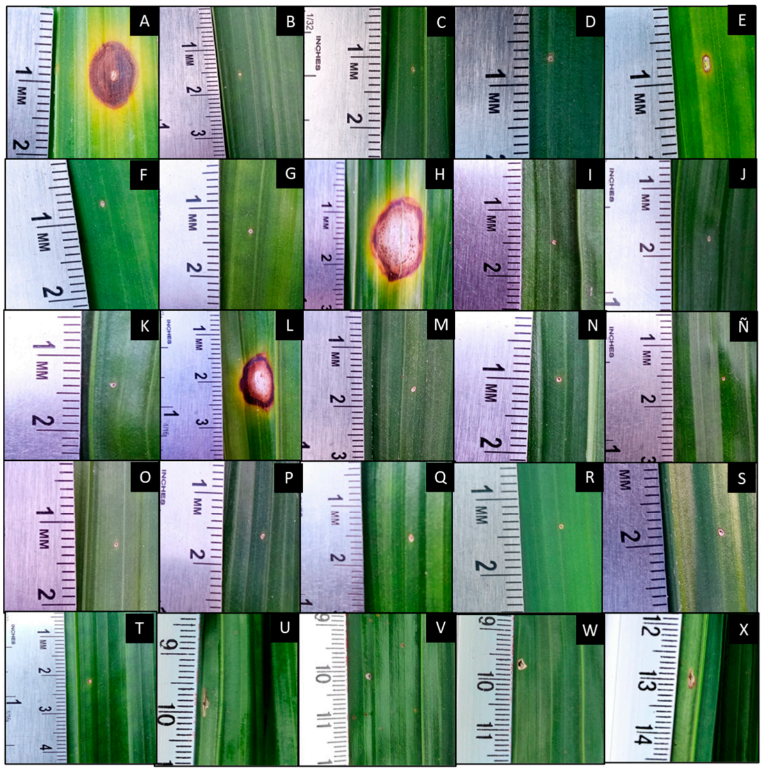

A	MFTU01-1
B	MFTU04-3
C	MFTU06-1
D	MFTU06-2
E	MFTU06-3
F	MFTU07-1
G	MFTU07-2
H	MFTU12
I	MFTU14-1
J	MFTU14-2
K	MFTU18
L	MFTU21
M	MFTU25
N	MFTU34-2
Ñ	MFTU34-3
O	MFTU35
P	MFTU36
Q	MFTU37
R	MFTU38
S	MFTU39-1
T	MFTU39-2
U	MFTU40-1
V	MFTU41
W	MFTU43
X	MFTU44-2

Supplementary Figure S6. Pathogenicity test of the 25 isolates related to *Pestalotiopsis* in cultivar Coarí x La Mé. The photos were taken 21 days after inoculation, representing isolates A-X as listed in Table 1.

Pathogenicity test of 25 isolates on the Manaos x Compacta cultivar.

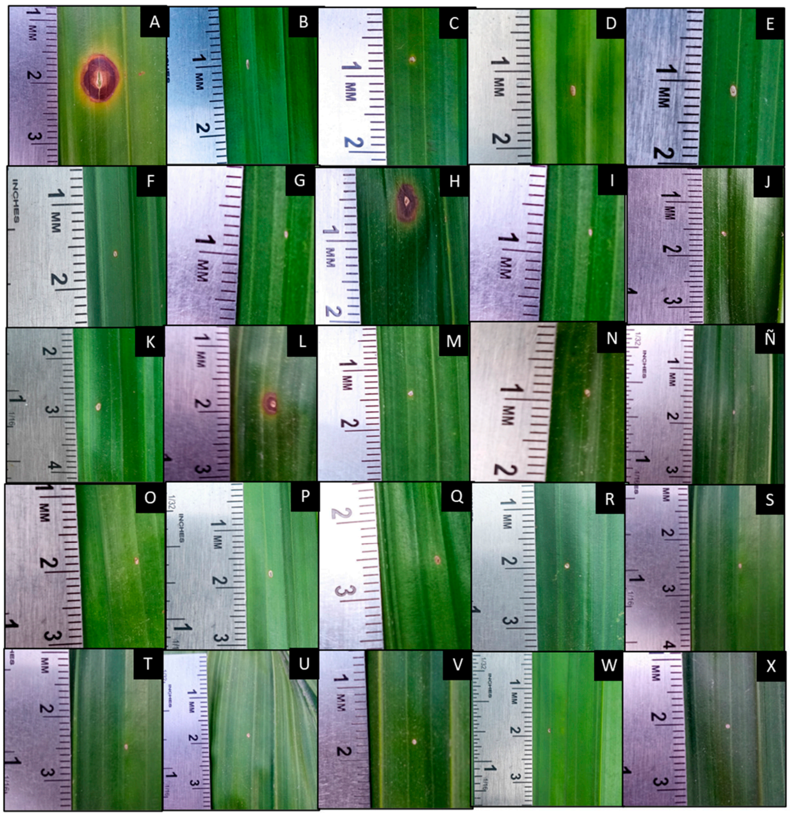

**A**	MFTU01-1
**B**	MFTU04-3
**C**	MFTU06-1
**D**	MFTU06-2
**E**	MFTU06-3
**F**	MFTU07-1
**G**	MFTU07-2
**H**	MFTU12
**I**	MFTU14-1
**J**	MFTU14-2
**K**	MFTU18
**L**	MFTU21
**M**	MFTU25
**N**	MFTU34-2
**Ñ**	MFTU34-3
**O**	MFTU35
**P**	MFTU36
**Q**	MFTU37
**R**	MFTU38
**S**	MFTU39-1
**T**	MFTU39-2
**U**	MFTU40-1
**V**	MFTU41
**W**	MFTU43
**X**	MFTU44-2

Supplementary Figure S7. Pathogenicity test of the 25 isolates related to *Pestalotiopsis* in cultivar Manaos x Compacta. The photos were taken 21 days after inoculation, representing isolates A-X as listed in Table 1.

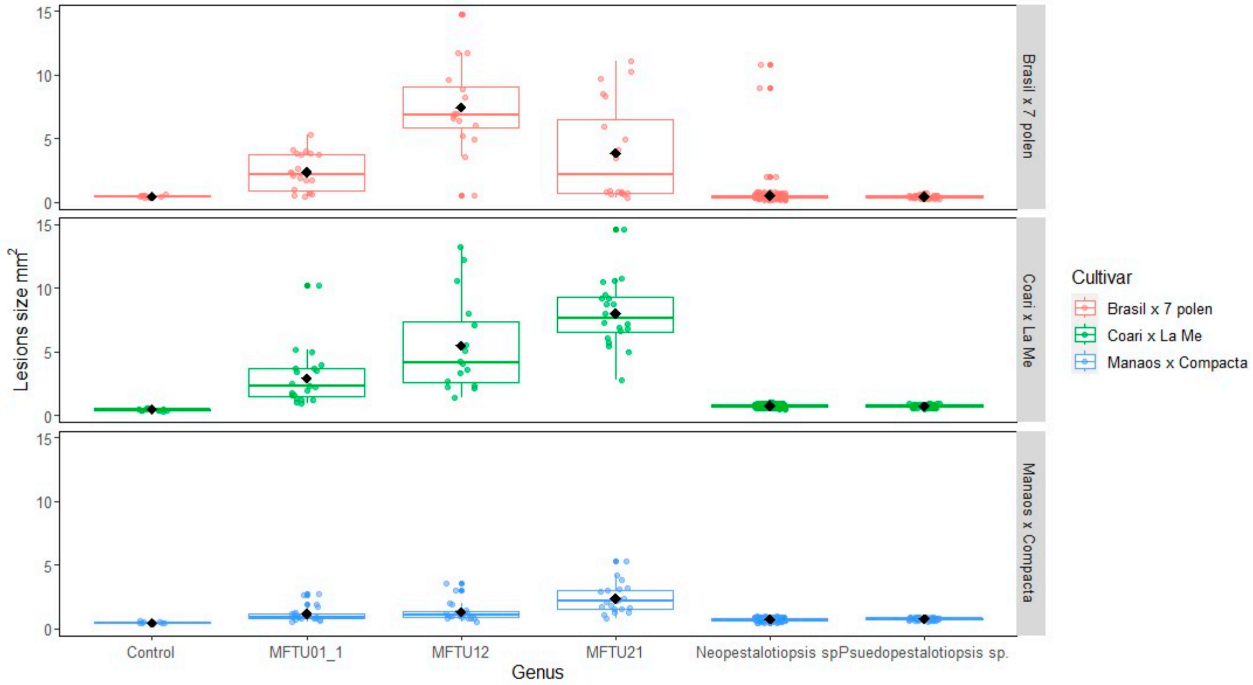

Supplementary Figure S8. The average size of lesions induced via inoculation with isolates of the genera *Pestalotiopsis*, *Neopestalotiopsis*, and *Pseudopestalotiopsis* in the cultivars (Brazil x 7 pollen africans, Coari x La Mé, and Manaos x Compacta). ANOVA and comparisons between treatments were conducted using Tukey's test ($\alpha < 0.05$).
